# Previous preeclampsia and its association with the future development of cardiovascular diseases: a systematic review and meta-analysis

**DOI:** 10.6061/clinics/2021/e1999

**Published:** 2021-01-11

**Authors:** Eduardo Carvalho de Arruda Veiga, Paulo Ricardo Higassiaraguti Rocha, Leonardo L. Caviola, Viviane Cunha Cardoso, Fabricio da Silva Costa, Maria da Conceição Pereira Saraiva, Marco Antonio Barbieri, Heloisa Bettiol, Ricardo Carvalho Cavalli

**Affiliations:** I Departamento de Obstetricia e Ginecologia, Hospital Universitario, Faculdade de Medicina de Ribeirao Preto, Universidade de Sao Paulo (FMRP-USP), SP, BR; II Departamento de Puericultura e Pediatria, Faculdade de Medicina de Ribeirao Preto, Universidade de Sao Paulo (FMRP-USP), SP, BR; III Disciplina de Ginecologia, Departamento de Obstetricia e Ginecologia, Hospital Universitario, Faculdade de Medicina de Ribeirao Preto, Universidade de Sao Paulo (FMRP-USP), SP, BR; IV Departamento de Odontologia Pediatrica, Escola de Odontologia de Ribeirao Preto, Faculdade de Medicina de Ribeirao Preto, Universidade de Sao Paulo (FMRP-USP), SP, BR

**Keywords:** Preeclampsia, Future Cardiovascular Diseases, Systematic Review, Meta-Analysis

## Abstract

Preeclampsia is a multifactorial disease. Among these factors, untreated hypertension during pregnancy can result in high morbidity and mortality rates and may also be related to the future development of cardiovascular diseases.Therefore, this systematic review aimed to determine the association of previous preeclampsia with the future development of cardiovascular diseases.

Studies on the association between preeclampsia and future cardiovascular diseases published in the last 10 years (2009-2019) were identified from the PubMed/Medline (207 articles), Embase (nine articles), and Cochrane (three articles) databases using the keywords “preeclampsia” and “future cardiovascular diseases”, “preeclampsia” and “future heart attack”, and “preeclampsia” and “future cardiac disease”. After applying the inclusion and exclusion criteria, 15 articles were analyzed by systematic review and meta-analysis according to the Preferred Reporting Items for Systematic Reviews and Meta-Analyses (PRISMA) guidelines. The meta-analysis and the determination of the quality of the articles were conducted using RevMan software, version 5.3.

Statistically significant differences were observed between the control and previous preeclampsia groups with respect to systolic blood pressure (mean difference [MD] 4.32; 95% confidence interval [95%CI] 3.65, 4.99; *p*<0.001), diastolic blood pressure (MD): 2.11; 95%CI: 1.68, 2.55; *p*<0.0001), and insulin level (MD: 2.80; 95% CI: 0.50, 5.11; *p*<0.001). Body mass index (MD: 2.57, 95%CI: 2.06, 3.07; *p*=0.0001), total cholesterol (MD: 10.39; 95%CI: 8.91, 11.87; *p*=0.0001), HDL (MD: 2.83; 95%CI: 2.20, 3.46; *p*=0.0001), and LDL (MD: 1.77; 95%CI: 0.42, 3.13; *p*=0.0001) also differed significantly between groups.

Thus, the results of the present study showed that women with a history of preeclampsia were more likely to develop cardiovascular disease.

## INTRODUCTION

Preeclampsia (PE) is defined as a pregnancy complication characterized by high blood pressure and signs of damage to other organs, with dysfunctions in the circulatory, renal, hematological, hepatic, and neurological systems. PE usually begins after 20 weeks of pregnancy in women with previously normal blood pressure levels (1,2). This condition has high morbidity and mortality, affects 2-5% of pregnant women and is responsible for thousands of deaths of mothers and fetuses worldwide (3).

The risk factors for the development of PE include preexisting diabetes, pre-pregnancy body mass index ≥35 kg/m^2^, nulliparity, family history of PE, twin pregnancy, maternal age ≥40 years, systolic blood pressure ≥130 mmHg, chronic hypertension, history of chronic kidney disease, and assisted reproductive technologies (4,5). Among the pathophysiological mechanisms of PE development is vascular dysfunction, defined as an imbalance between pro-angiogenic and anti-angiogenic factors. The levels of markers such as vascular endothelial growth factor and placental growth factor (PIGF) are increased in endothelial vascular dysfunction, with a consequent increase in systolic and diastolic pressures and proteinuria in pregnant women (3,6).

While previous studies have demonstrated the association of PE development in pregnant women and future cardiovascular complications such as the development of hypertension, diabetes, ischemic disease, and other cardiovascular diseases, recent literature has suggested the need for additional studies for a more complete understanding of how this relationship is established (7-11).

Therefore, the objective of the present systematic review and meta-analysis was to evaluate the association between previous PE and future cardiovascular diseases.

## MATERIAL AND METHODS

The literature search strategy was applied as recommended by Berstock et al. (2019) (12). We searched for articles published from January 2009 to July 2020. This time interval was chosen because the diagnosis of PE has improved and changed in the last decade. We first selected keywords from related articles and used Medicals Subject Headings (MeSH) to identify more related keywords with close meaning, such as: (“preeclampsia” [MeSH Terms] OR (“future cardiovascular diseases” [All Fields], as well as the MeSH terms “preeclampsia and future heart attack” and &quot;preeclampsia and future cardiac disease” The search strategy was applied to the PubMed/Medline, Embase, and Cochrane databases.

This review was conducted according to the recommendations established by the Preferred Reporting Items for Systematic Reviews and Meta-Analysis (PRISMA) (13,14).

Two researchers with the ability to compile systematic reviews (E.C.V. and P.R.H.R.) independently and blindly retrieved the papers and evaluated the titles and abstracts with reference to the inclusion and exclusion criteria according to the PICO components (14). The selected articles were then critically evaluated for inclusion or exclusion in the review. Disagreements between the investigators regarding the inclusions of studies were resolved following consultation with a third reviewer (L.L.C.).

The main outcomes were increased risks of diabetes mellitus, dyslipidemia, hypertension, cardiovascular heart failure, and brain disease, as well as an increased risk of cardiac diastolic dysfunction. The other outcomes were increased risks of mother and child mortality, stroke, ischemic heart disease, increased risk of coronary artery calcification, greater relative wall thickness, and reduced left ventricular end-diastolic volume (Table 1). These qualitative results are the descriptors of various types of cardiovascular diseases that women with a history of PE eventually develop. Among several cardiovascular diseases that can be assessed, the most common are described here.

RevMan 5.3 (Cochrane Collaboration, Oxford, UK) was used to perform the meta-analysis. A random-effects model was used for the assessment of heterogeneity.

### Statistical analysis

The mean values and standard deviation of the studies are presented as mean difference (MD) of the post-intervention values after calculating the inverse of the variance to determine the magnitude of the effect of PE and future cardiovascular diseases (15). Heterogeneity was assessed using the Cochran and I^2^ Q tests, followed by visual inspection of the graph.

Heterogeneity between studies was defined as an I^2^ value above 50% (16,17). The analyses were performed using RevMan 5.3.

## RESULTS

**Figure 1:**
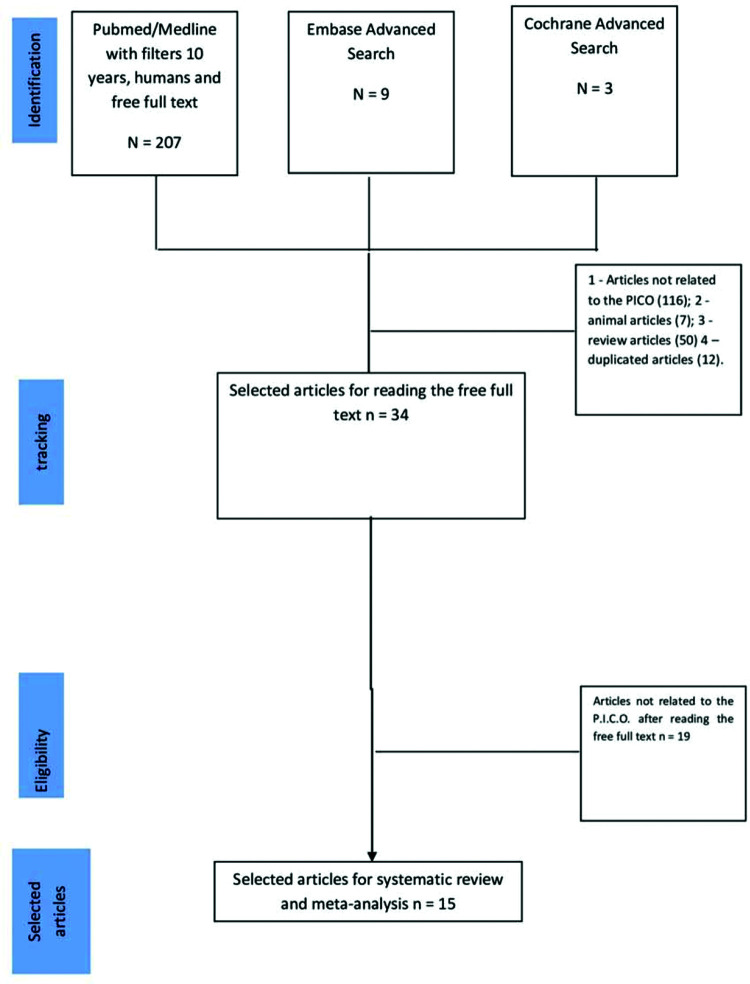
Flowchart showing the study selection.

The search process, identification, and selection of the articles are shown in Fig. 1. The inclusion criteria were articles published in English related to the keywords preeclampsia and future cardiovascular disease appearing in the titles and abstracts of the selected articles. Articles without these keywords were excluded because they were not related to the Patient/Problem/Population, Intervention, Comparison, and Outcome(s) (PICO) design for the study, as follows. Patients: women with PE, Intervention: classic definitions by the articles of women with PE and optimal medical treatment during pregnancy, Comparator: women without PE and with pregnancy without hypertension, and Outcomes: future cardiovascular diseases in by the target audience (13). The exclusion criteria in the PubMed database were the filters “last 10 years”, “humans”, and “free full text”. This search identified 207 articles. We also searched for the keywords with an advanced search of the titles, which identified nine and three articles in the Embase and Cochrane databases, respectively. The next step was to apply the following exclusion criteria: we excluded 116 articles not related to the PICO, seven articles on animals, 50 review articles, and 12 duplicate articles. Of 34 articles selected for complete text review, 19 were excluded for not being related to the PICO. Thus, a total of 15 articles were finally selected for inclusion in the systematic review and meta-analysis (Fig. 1).

The information obtained from the studies selected for the systematic review is presented in Table 1, which lists the following characteristics of the articles: authors, year of publication, country, baseline, study years, study design, definition of PE according to systolic and diastolic blood pressures (SBP and DBP, respectively), number of participants in the study, follow-up time (mean and range in years), age at follow-up (mean and range in years), and outcomes.

Among the cardiovascular diseases that developed after PE that were reported in our selected articles, the most prevalent was hypertension (18-26,30-32), followed by diabetes (18,20,21,24-28,30,31), and heart failure (18,19,22,23,26,27,30,31). Stroke and brain diseases also occurred in some patients a few years after the incidence of PE (18,23,24,27,31), as well as coronary artery calcification in some women (21,29) (Table 2). It is important to emphasize that the moment of the evaluations of the variables of the meta-analysis in the selected articles were made after the follow-up, either one year or ten years or more after the diagnosis of PE, so that we can have as an outcome and as a result, the appearance of cardiovascular diseases.

**Figure 2:**
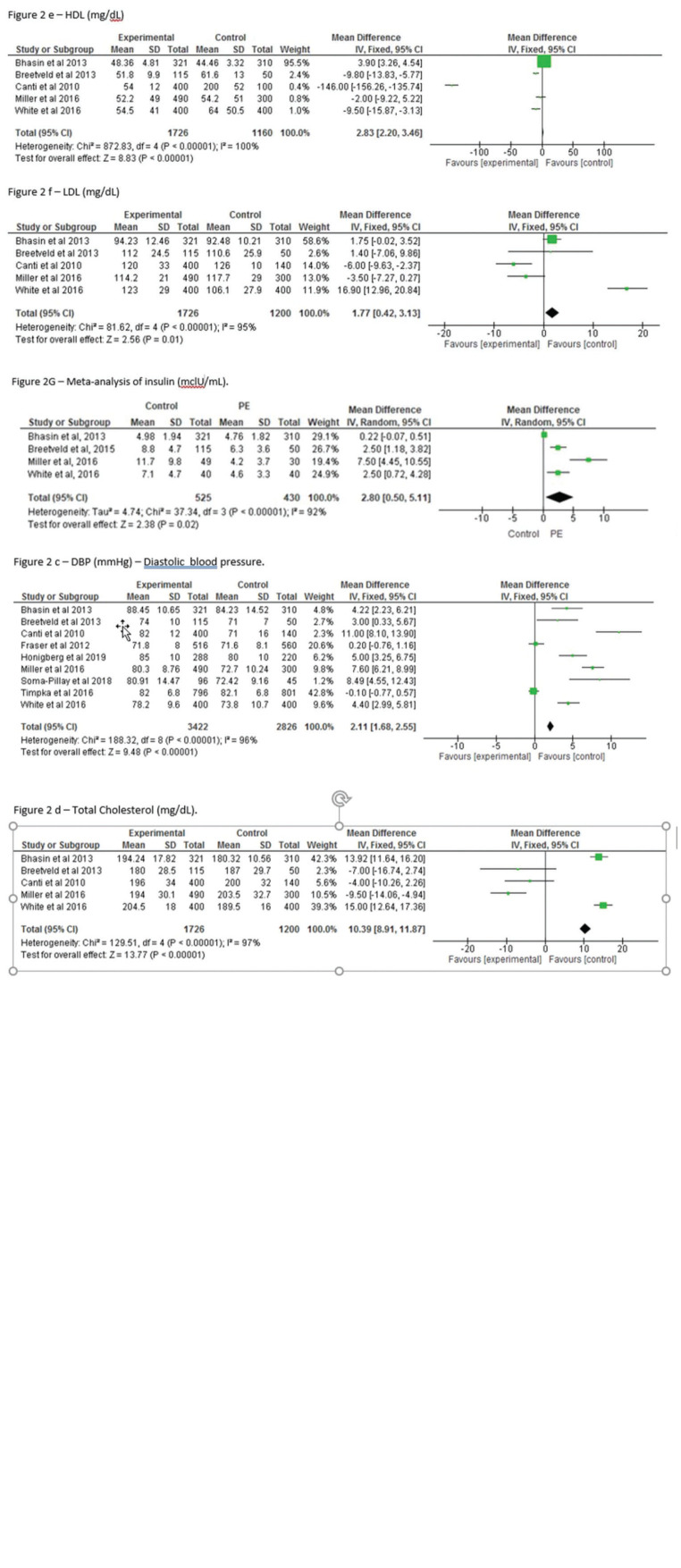
A-G, Meta-analysis of the relationships between body mass index, systolic blood pressure, diastolic blood pressure, total cholesterol, high-density lipoprotein, low-density lipoprotein, and insulin levels and the later development of cardiovascular diseases according to the history of preeclampsia.

The results of the meta-analysis are described below. Comparison of body mass index (BMI) between the PE and control groups showed a significantly higher value in the PE group, indicating that women with previous PE were heavier than the control women (MD: 2.57, 95% confidence interval [95%CI]: 2.06, 3.07; *p*≤0.0001) (Fig. 2A). As expected, SBP was significantly higher among women in the previous PE group than that in the control group (MD: 4.32; 95%CI: 3.65, 4.99; *p*<0.00001) (Fig. 2B). Moreover, as expected, DBP was also significantly higher among women with previous PE (MD: 2.11; 95%CI: 1.68, 2.55, *p*<0.00001) compared to that in the control groups. In addition, the CI between groups was low (Fig. 2C). Blood insulin also differed significantly between women with PE and the controls (MD: 2.80; 95%CI: 0.50, 5.11; *p*<0.00001) (Fig. 2G).

We also observed statistically significant differences in total cholesterol level (MD: 10.39; 95%CI: 8.91, 11.87; *p*<0.0001) (Fig. 2D), high-density lipoprotein (HDL) level (with a broad confidence interval) (MD: 2.83; 95%CI: 2.20, 3.46; *p*≤0.0001) (Fig. 2E), and low-density lipoprotein level (LDL) (MD: 1.77; 95%CI: 0.42, 3.13; *p*=0.09) (Fig. 2F).

## DISCUSSION

The major limitation of this study was that most of the studies included in the systematic review followed the mothers for less than 10 years, a fact that only allowed the analysis of blood markers and not the development of cardiovascular disease in women with a history of PE. Another limitation of this study was that, in bias risk analysis, the variables related to the blinding of the participants and personnel, blinding of the outcome assessment, and other biases showed unclear risks of bias. However, the strength of this study was that it demonstrated increased levels of cardiovascular disease variables in women with PE, indicating the probable future development of cardiovascular diseases.

The main findings of the present meta-analysis were increased BMI, SBP, DBP, HDL, LDL, total cholesterol, and insulin concentration in the previous PE group compared to those in the control group.

Among the articles included in the systematic review (18-32), we identified several outcomes leading to the increased incidence of future cardiovascular disease among women with PE, including increased mortality and two-fold increases in diabetes, hypertension, myocardial infarction, and other cardiovascular diseases. The results of these studies were concordant with those of four recent meta-analyses (33-35) showing that women with PE have higher chances of developing future cardiovascular diseases as well as a higher risk of mortality. One meta-analysis reported higher chances of developing coronary heart disease, stroke, and death due to cardiovascular disease (33); another reported higher chances of developing hypertension, ischemic heart disease, heart failure, and cerebrovascular accidents (34). Alma et al. (2017) (33) analyzed the role of biomarkers such as C-reactive protein, HDL, and insulin as markers of future cardiovascular diseases and observed increased levels in women with PE compared to those in pregnant women without increased blood pressure. In a study of women with hypertensive diseases in pregnancy, Ukah et al. (2018) (35) used multivariate models to demonstrate that these women are at higher risk of developing cardiovascular disease in the future. Irgens et al. (2001) (38) assessed whether mothers and fathers were at higher risk of death and cardiovascular disease after the mother was diagnosed with PE and concluded that genetic factors increased the chance of developing cardiovascular risks. Sattar et al. (2002) (39) discussed the pre-gestational metabolic status of women, suggesting that an adverse pregnancy increases the risk of future vascular and metabolic diseases.

Our meta-analysis identified the following six significant and consistent factors among PE patients: higher SBP (19,21-23,25,26,29), higher DBP (19,21-23,25,26,29), higher blood insulin value, which is indicative of future diabetes development (21,23,25,29); and higher HDL, LDL, and insulin levels. These results agree in part with those in recent literature, including the meta-analyses by de Groot et al. (2017) (33) and Brouwers et al. (2018) (34). However, most systematic meta-analyses have investigated other parameters in women with PE or with hypertensive disease in pregnancy; that is, the development of various cardiovascular diseases such as the risks of heart failure, coronary heart disease, and death from cardiovascular disease, focusing on different variables from those analyzed in the present study (32). Other studies performed different analyses; for example, Alma et al. (2017) (33) reported higher C-reactive protein and HDL levels in women with PE compared to those in controls. Ukah et al. (2018) (35) used another approach comparing the quality of assessment and accuracy and specificity in women with PE and other hypertensive diseases in pregnancy.

In our meta-analysis, BMI (19,21-23,25,26,29,31,32) showed statistical significance (*p*≤0.0001), consistent with recent findings (3,32) reporting a two-fold increase in the risk of diabetes in women with PE, as well as increased hypertension and serum glucose and lipid levels. Total cholesterol (21,23,25,29,31,32) and LDL (21,23,25,29,31,32) concentrations were higher in the control group, although the differences were not statistically significant. Women with a history of PE had significantly higher HDL levels (21,23,25,29,31,32). However, these relationships varied in the literature depending on socioeconomic and quality of life factors such as age, smoking, alcohol consumption, and physical activity (36,37).

## CONCLUSION

In conclusion, the results of this study showed that women with previous PE were more likely to develop cardiovascular disease in the future.

## AUTHOR CONTRIBUTIONS

de Arruda Veiga EC participated with substantial contributions to study concept and design, intellectual content, literature search, data and statistical analysis, manuscript preparation, article drafting, critical revision for important intellectual content and final approval of the manuscript. Rocha PR participated with literature search, data and statistical analysis, manuscript preparation, article drafting or critical revision for important intellectual content and final approval of the version to be published. Bettiol H, Barbieri MA, Caviola LL and da Conceição Pereira Saraiva M participated with data and statistical analysis, article drafting or critical revision for important intellectual content and final approval of the version to be published. Cardoso VC participated with data and statistical analysis, article drafting or critical revision for important intellectual content final approval of the version to be published.FSC-Data and statistical analysis, article drafting or critical revision for important intellectual content and final approval of the version to be published. Cavalli RC participated with substantial contributions to study concept, design and definition of intellectual content, article drafting and critical revision for intellectual content and final approval of the version to be published.

## Figures and Tables

**Table 1:** Characteristics of studies about women with preeclampsia.

Author and year of publication	Country, baseline years of study	Study design	Definition of preeclampsia (SBP, DBP)	No. of participants	Follow-up time (median, range, years)	Age at follow-up (median, range, years)	Outcome
Gastrich et al. 2020	USA	Case-control study	NS	6,360	15	26.78	Cardiac diseases, diabetes, stroke, myocardial infarction.
Honigberg et al. 2019	USA	Observational study	SBP≥140 and/or DBP≤90 mmHg	2,808	7	57.4	Hypertension, hyperlipidemia, diabetes mellitus.
Kuo et al. 2018	Taiwan 1996-2010	Retrospective longitudinal study	NS	6,475	9.8	30.5	Increased risks of DM, dyslipidemia, hypertension, CHF, and cerebrovascular diseases.
Soma-Pillay et al. 2018	South Africa 2013-2016	Descriptive study	Mean (SD) 128 (14.17) -115 (9.89)	141	1	28.9	Increased risk of cardiac diastolic dysfunction one year after delivery.
Theilen et al. 2018	USA 1939-2012	Retrospective cohort study	NS	172,152	18	-	Increased risks of mortality from DM, ischemic heart disease, and stroke.
Parikh et al. 2017	Sweden 1973-2011	Prospective cohort study	SBP≥140 and/or DBP≤90 mmHg	16,009	37	40	Women presenting with preeclampsia, gestational diabetes, or placental abruption.
Miller et al. 2016	USA	Case-control study	NS	79	1	60	Women with histories of preeclampsia, both waist circumference and measures of insulin resistance were greater in women with coronary artery calcification.
Timpka et al. 2016	Sweden, USA, UK 1991-1992	Prospective birth cohort study	≥140, ≤90	1,511	1	17	Greater relative wall thickness and reduced left ventricular end-diastolic volume.
White et al. 2016	USA 1976-1982	Cohort	≥140, ≤90	80	6	59	Among women with histories of preeclampsia, the presence of coronary artery calcifications.
Breetveld et al. 2015	Netherlands 2010-2012	Observational study	≥140, ≤90	115	3	25-45	A two-fold risk of developing cardiovascular disease.
Savitz et al. 2014	USA 1995-2004	Observational study	NS	849,639 births	1	1	Hypertensive disorders of pregnancy strongly predicted short-term risk of hospitalization for chronic disease within 1 year of delivery,
Bhasin et al. 2013	India	Observational study	NS	631	10	33	Hypertension pregnancy, DM, and pregnancy outcomes were all associated with an increased risk of CVD 10 years later
Feig et al. 2013	Canada 1994-2008	Retrospective cohort study	NS	1,010,068	16	15-50	Preeclampsia was associated with a two-fold increased incidence of diabetes.
Frasier et al. 2012	United Kingdom	Prospective cohort study	≥140, ≤90	3,416	18	Mean 48	Both gestational hypertension and preeclampsia were associated with a greater number of cardiovascular risk factors.
Canti et al. 2010	BR	Cross-sectional study	NS	40	10	NS	Blood pressure measurements.

Legends: DM - diabetes mellitus; CVD - cardiovascular disease; CHF - congestive heart failures.

**Table 2:** Studies including women with PE and increased risks of the development of several cardiovascular diseases in general or specifically.

Authors	Cardiovascular disease in general	*Diabetes mellitus*	Heart failure	Stroke and cerebral diseases	Coronary artery calcification	Hypertension
Gastrich et al. 2020	X	*X*	X	X		X
Honigbert et al. 2019	X		X		X	X
Kuo et al. 2018	X	X	X	X	-	X
Som sa-Pillay et al. 2018	X	-	X	-	-	X
Theilen et al. 2018	X	X	X	X	-	-
Parikh et al. 2017	X	X	-	-	-	X
Miller et al. 2016	X	X	-	-	X	X
Timpka et al. 2016	X	-	X	-	-	X
White et al. 2016	X	-	-	-	X	-
Breetveld et al. 2015	X	-	X	X	-	X
Savitz et al. 2014	X	X	-	X	-	X
Bhasin et al. 2013	X	X	-	-	-	X
Feig et al. 2013	-	X	-	-	-	-
Frasier et al. 2012	X	X	X	-	-	X
Canti et al. 2010	X					X

X: relationship between women with preeclampsia and the future development of various types of cardiovascular disease.

-: no specific mention of this cardiovascular disease.

## References

[1] Ahmed A, Rezai H, Broadway-Stringer S (2017). Evidence-Based Revised View of the Pathophysiology of Preeclampsia. Adv Exp Med Biol.

[2] Phipps E, Prasanna D, Brima W, Jim B (2016). Preeclampsia: Updates in Pathogenesis, Definitions, and Guidelines. Clin J Am Soc Nephrol.

[3] Rana S, Lemoine E, Granger JP, Karumanchi SA (2019). Compendium on the pathophysiology and treatment of hypertension. Cir Res.

[4] Burton GJ, Redman CW, Roberts JM, Moffett A (2019). Pre-eclampsia: pathophysiology and clinical implications. BMJ.

[5] Rabaglino MB, Conrad KP (2019). Evidence for shared molecular pathways of dysregulated decidualization in preeclampsia and endometrial disorders revealed by microarray data integration. FASEB J.

[6] Tomimatsu T, Mimura K, Matsuzaki S, Endo M, Kumasawa K, Kimura T (2019). Preeclampsia: Maternal Systemic Vascular Disorder Caused by Generalized Endothelial Dysfunction Due to Placental Antiangiogenic Factors. Int J Mol Sci.

[7] Gamble DT, Brikinns B, Myint PK, Bhattacharya S (2019). Hypertensive Disorders of Pregnancy and Subsequent Cardiovascular Disease: Current National and International Guidelines and the Need for Future Research. Front Cardiovasc Med.

[8] Benschop L, Schalekamp-Timmermans S, Broere-Brown ZA, Roeters van Lennep JE, Jaddoe VWV, Roos-Hesselink JW (2019). Placental Growth Factor as an Indicator of Maternal Cardiovascular Risk After Pregnancy. Circulation.

[9] Cooke CM, Davidge ST (2019). Advanced maternal age and the impact on maternal and offspring cardiovascular health. Am J Physiol Heart Circ Physiol.

[10] Kazmi N, Sharp GC, Reese SE, Vehmeijer FO, Lahti J, Page CM (2019). Hypertensive disorders of pregnancy and DNA methylation in newborns Hypertension.

[11] Lane-Cordova AD, Khan SS, Grobman WA, Greenland P, Shah SJ (2019). Long-Term Cardiovascular Risks Associated With Adverse Pregnancy Outcomes: JACC Review Topic of the Week. J Am Coll Cardiol.

[12] Berstock JR, Whitehouse MR (2019). How to prepare and manage a systematic review and meta-analysis of clinical studies. EFORT Open Rev.

[13] Moher D, Shamseer L, Clarke M, Ghersi D, Liberati A, Petticrew M (2015). Preferred reporting items for systematic review and meta-analysis protocols (PRISMA-P) 2015 statement. Syst Rev.

[14] Zorzela L, Loke YK, Ioannidis JP, Golder S, Santaguida P, Altman DG (2016). PRISMA harms checklist: improving harms reporting in systematic reviews.. BMJ.

[15] DerSimonian R, Kacker R (2007). Random-effects model for meta-analysis of clinical trials: an update. Contemp Clin Trials.

[16] von Hippel PT (2015). The heterogeneity statistic I(2) can be biased in small meta-analyses. BMC Med Res Methodol.

[17] Higgins JP, Thompson SG (2002). Quantifying heterogeneity in a meta-analysis. Stat Med.

[18] Kuo YL, Chan TF, Wu CY, Ker CR, Tu HP (2018). Preeclampsia-eclampsia and future cardiovascular risk among women in Taiwan. Taiwan J Obstet Gynecol.

[19] Soma-Pillay P, Louw MC, Adeyemo AO, Makin J, Pattinson RC (2018). Cardiac diastolic function after recovery from pre-eclampsia. Cardiovasc J Afr.

[20] Parikh NI, Norberg M, Ingelsson E, Cnattingius S, Vasan RS, Domellöf M (2017). Association of Pregnancy Complications and Characteristics With Future Risk of Elevated Blood Pressure: The Västerbotten Intervention Program. Hypertension.

[21] Miller VM, Garovic VD, Bailey KR, Lahr BD, Mielke MM, White WM (2016). Pregnancy history and blood-borne microvesicles in middle aged women with and without coronary artery calcification. Atherosclerosis.

[22] Timpka S, Macdonald-Wallis C, Hughes AD, Chaturvedi N, Franks PW, Lawlor DA (2016). Hypertensive Disorders of Pregnancy and Offspring Cardiac Structure and Function in Adolescence. J Am Heart Assoc.

[23] Breetveld NM, Ghossein-Doha C, van Kuijk S, van Dijk AP, van der Vlugt MJ, Heidema WM (2015). Cardiovascular disease risk is only elevated in hypertensive, formerly preeclamptic women. BJOG.

[24] Savitz DA, Danilack VA, Elston B, Lipkind HS (2014). Pregnancy-induced hypertension and diabetes and the risk of cardiovascular disease, stroke, and diabetes hospitalization in the year following delivery. Am J Epidemiol.

[25] Bhasin P, Kapoor S (2014). Pregnancy complications and calculated cardiovascular risk in urban women: do we envisage an association?. J Urban Health.

[26] Fraser A, Nelson SM, Macdonald-Wallis C, Cherry L, Butler E, Sattar N (2012). Associations of pregnancy complications with calculated cardiovascular disease risk and cardiovascular risk factors in middle age: the Avon Longitudinal Study of Parents and Children. Circulation.

[27] Theilen LH, Meeks H, Fraser A, Esplin MS, Smith KR, Varner MW (2018). Long-term mortality risk and life expectancy following recurrent hypertensive disease of pregnancy. Am J Obstet Gynecol.

[28] Feig DS, Shah BR, Lipscombe LL, Wu CF, Ray JG, Lowe J (2013). Preeclampsia as a risk factor for diabetes: a population-based cohort study. PLoS Med.

[29] White WM, Mielke MM, Araoz PA, Lahr BD, Bailey KR, Jayachandran M (2016). A history of preeclampsia is associated with a risk for coronary artery calcification 3 decades later. Am J Obstet Gynecol.

[30] Gastrich MD, Zinonos S, Bachmann G, Cosgrove NM, Cabrera J, Cheng JQ (2020). Preeclamptic Women Are at Significantly Higher Risk of Future Cardiovascular Outcomes Over a 15-Year Period. J Womens Health (Larchmt).

[31] Honigberg MC, Zekavat SM, Aragam K, Klarin D, Bhatt DL, Scott NS (2019). Long-Term Cardiovascular Risk in Women With Hypertension During Pregnancy. J Am Coll Cardiol.

[32] Canti IC, Komlós M, Martins-Costa SH, Ramos JG, Capp E, Corleta Hv (2010). Risk factors for cardiovascular disease ten years after preeclampsia. Sao Paulo Med J.

[33] Alma LJ, Bokslag A, Maas AHEM, Franx A, Paulus WJ, de Groot CJM (2017). Shared biomarkers between female diastolic heart failure and pre-eclampsia: a systematic review and meta-analysis. ESC Heart Fail.

[34] Brouwers L, van der Meiden-van Roest AJ, Savelkoul C, Vogelvang TE, Lely AT, Franx A (2018). Recurrence of pre-eclampsia and the risk of future hypertension and cardiovascular disease: a systematic review and meta-analysis. BJOG.

[35] Ukah UV, De Silva DA, Payne B, Magee LA, Hutcheon JA, Brown H (2018). Prediction of adverse maternal outcomes from pre-eclampsia and other hypertensive disorders of pregnancy: A systematic review. Pregnancy Hypertens.

[36] Sapranaviciute-Zabazlajeva L, Luksiene D, Virviciute D, Bobak M, Tamosiunas A (2017). Link between healthy lifestyle and psychological well-being in Lithuanian adults aged 45-72: a cross-sectional study. BMJ Open.

[37] Kazemi Karyani A, Karmi Matin B, Soltani S, Rezaei S, Soofi M, Salimi Y (2019). Socioeconomic gradient in physical activity: findings from the PERSIAN cohort study. BMC Public Health.

[38] Irgens HU, Reisaeter L, Irgens LM, Lie RT (2001). Long term mortality of mothers and fathers after pre-eclampsia: population based cohort study. BMJ.

[39] Sattar N, Greer IA (2002). Pregnancy complications and maternal cardiovascular risk: opportunities for intervention and screening?. BMJ.

